# Study on the Effect of Structural Modification of Xanthan Gum on Its Synergistic Gelation Performance with Locust Bean Gum

**DOI:** 10.3390/molecules31101597

**Published:** 2026-05-10

**Authors:** Yusen Wu, Wei Wang, Yonggang Zhang, Yanmin Zhang, Siduo Zhou, Xueqian Dong

**Affiliations:** 1 Shandong Key Laboratory of Healthy Food Resources Exploration and Creation, School of Food Science and Engineering, Qilu University of Technology, Jinan 250353, China; 10431230868@stu.qlu.edu.cn (Y.W.); wangwei87@qlu.edu.cn (W.W.); zyg8500@qlu.edu.cn (Y.Z.); b1043125050@stu.qlu.edu.cn (Y.Z.); 2 Shandong Research and Design Institute of Food & Fermentation Industry, Qilu University of Technology, Jinan 250013, China

**Keywords:** xanthan gum, locust bean gum, polysaccharide structure, gel properties

## Abstract

The synergistic gelation between xanthan gum (XG) and locust bean gum (LBG) is a classic phenomenon widely adopted for quality control of XG functionality; yet the regulatory roles of XG’s side chain groups—particularly glucuronic acid, whose function remains unexplored—have not been systematically elucidated. In this study, three complementary modification strategies including enzymatic hydrolysis, oxalic acid treatment, and dilute alkali treatment were for the first time combined to precisely modulate the contents of pyruvate, acetyl, and glucuronic acid in XG side chains, constructing a series of XG samples with well-defined gradient structures. Enzymatic hydrolysis and oxalic acid treatment reduced the pyruvate content from 5.80% to 1.05% and 1.42%, respectively, while dilute alkali treatment selectively decreased the acetyl content from 3.97% to 2.58%. The effects were systematically investigated through multi-scale characterization including rheology, texture analysis, scanning electron microscopy and thermogravimetric analysis, combined with correlation analysis. The results revealed that glucuronic acid, together with pyruvate, synergistically enhanced gel network stability through electrostatic interactions and hydrogen bonding. In contrast, acetyl groups acted as negative regulators via steric hindrance, inhibiting hydrogen-bond crosslinking. This study clarifies the distinct functional roles of key XG side chain groups, with the first systematic demonstration of glucuronic acid’s contribution, and provides a theoretical basis for the structure-oriented precise design of XG-based functional gels.

## 1. Introduction

Xanthan gum (XG) is an extracellular anionic polysaccharide produced through fermentation by *Xanthomonas campestris*. Its main chain consists of β-1,4-D-glucose, whereas the side chain is composed of β-D-mannose-(1,4)-β-D-glucuronic acid-(1,2)-α-D-mannose linkages [1]. XG exhibits two distinct conformations in aqueous solutions, namely ordered and disordered conformations, which are influenced by various factors, such as temperature, pH, and ionic strength [2]. In the industry, XG is widely used as a thickener, emulsifier, shaping agent, stabilizer, and dispersant [3]. Studies have shown that the removal of mannose residues at the termini of XG side chains endows XG with novel properties [4]. Therefore, based on the unique molecular structure of XG, the development of enzymes capable of hydrolyzing its side chains to obtain modified XG is highly promising.

Although XG acts as a high-viscosity suspending agent and thickener, it cannot independently form gels. However, XG can have a strong synergistic effect with certain polysaccharides, such as tamarind and *Gleditsia sinensis* polysaccharide gums [5,6]. XG from different production batches and sources exhibits significant differences in synergistic gelation capabilities owing to structural variations, such as molecular weight and acetyl and pyruvate contents.

The fine structure of XG, including pyruvate and acetyl groups, significantly affects its chain conformation. A higher acetyl content is conducive to formation of the helical structure of XG, whereas pyruvate groups maintain stability of the ordered structure via electrostatic repulsion [7]. Previous studies have demonstrated that the structure of XG significantly influences its synergistic gelation with other polysaccharides. Tako et al. [8] confirmed that the deacetylation of XG can slightly improve its gelation behavior, enabling the mixed gel to have a higher dynamic modulus. Liu et al. [5] reported that adjusting the fine structure of XG to increase its disordered structure enhances synergistic gelation with G. sinensis polysaccharide gum, whereas increasing its ordered structure inhibits this synergistic effect.

Compared with chemical modification, enzyme modification can specifically alter the fine structure of XG. XG lyase releases terminal mannose from the XG side chain through elimination, thereby generating tetrasaccharide repeating units and exposing glucuronic acid residues in the side chain [9]. XG lyase can be used in combination with other enzymes for the degradation of drilling fluids containing XG and production of XG oligosaccharides [10]. Our preliminary experiments revealed that the removal of terminal mannose and pyruvate significantly affects the synergistic gelation between XG and locust bean gum (LBG); however, the specific mechanism involved remains unclear. Additionally, the role glucuronic acid plays in synergistic XG gelation has rarely been explored.

LBG is a neutral, non-gelling polysaccharide extracted from seeds of the locust bean tree (*Ceratonia siliqua*). It consists of β-(1-4)-mannose main chains and α-(1-6)-galactose side chains, with a galactose-to-mannose ratio of approximately 1:4. In aqueous solutions, LBG can form a unique three-dimensional network structure that exhibits excellent thickening properties [11]. Furthermore, LBG can form intermolecular bonds with XG through hydrogen bonds and van der Waals forces, creating stable connections and thereby endowing the mixed solution with strong gelation properties [12,13]. Therefore, in China’s XG national standard, GB1886.41-2015, the synergistic gel effect of XG and LBG was adopted as a practical quality-control method to effectively evaluate the intrinsic functional quality of XG. Furthermore, LBG is a neutral polysaccharide with no self-standing gel-forming ability [14], which can minimize the interference from exogenous charged groups apart from those inherent to XG. Thus, this XG/LBG synergistic system allows for a clearer elucidation of the relationship between the fine structural features of XG and the functional performance of the corresponding synergistic gel.

Many hypotheses have been proposed concerning the synergistic binding sites or modes between LBG and XG, primarily focusing on their macromolecular interactions [15]. However, the influence that the fine structure of XG has on the synergistic gelation of XG and LBG has yet to be fully elucidated. Specifically, there is a lack of systematic dissection of the distinct functional roles of XG’s fine structural moieties (i.e., pyruvate and acetyl groups) in the synergistic gel; in addition, the role of glucuronic acid in the XG/LBG composite gel and the systematic effects of enzymatic modification on gel performance have rarely been reported.

**Figure 1:**
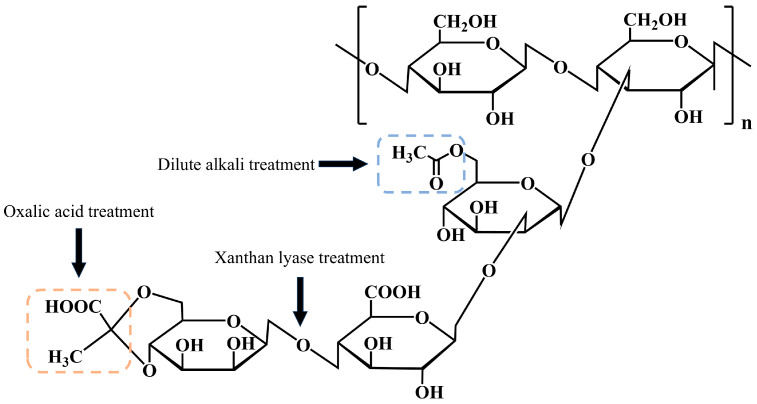
Conceptual diagram of xanthan gum side-chain modifications.

In this study, for the first time, three strategies including enzymatic hydrolysis, oxalic acid treatment, and dilute alkali treatment were adopted to actively and precisely tailor the acetyl groups, pyruvate groups, and pyruvated mannose units in the side chains of XG, constructing a series of XG samples with well-defined gradient contents of side chain groups and controllable structural differences. The specific modification sites are illustrated in Figure 1. Then, the chemical structures and conformational differences in various XG samples were characterized by determining their viscosity, molecular weight, and monosaccharide composition. A rheometer was employed to reveal their synergistic gelation process, whereas a texture analyzer, thermogravimetric (TG) analyzer, scanning electron microscope, low-field nuclear magnetic resonance (LF-NMR) analyzer, together with zeta potential measurement, Fourier transform infrared (FT-IR) spectroscopy, and X-ray diffraction (XRD) were used to comprehensively verify the effect that the fine XG structure has on the composite gel properties. Furthermore, a complete logical chain for the regulation of gel properties by the fine side chain structure of XG was established via correlation analysis, and the distinct functional roles of acetyl groups, pyruvate groups, and glucuronic acid in the synergistic gel were clarified.

This study provides suitable methods that could be used to further investigate the synergistic gelation between XG and polysaccharides and offers a theoretical basis for the design and production of composite gels based on XG-derived polysaccharides, as well as a theoretical foundation and regulatory pathway for the structure-oriented precise design of Xanthan gum-based functional composite gels with tailored molecular structures and targeted functional properties.

## 2. Results and Discussion

### 2.1. Expression and Purification of Xanthan Lyase

**Figure 2:**
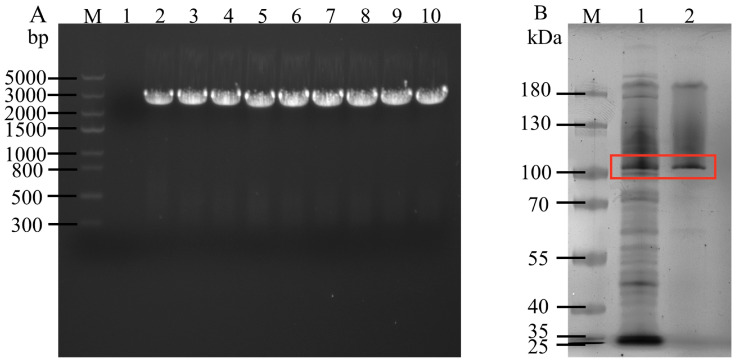
The electrophoresis results of the recombinant strain (**A**) and SDS-PAGE results of the enzyme solution (**B**). In (**A**), Lane M is the marker, Lane 1 is the negative control, Lane 2 is the positive control, and lanes 3 to 10 are the positive clones (target gene fragment). In (**B**), Lane M is the marker, Lane 1 is the crude enzyme solution, and Lane 2 is the purified enzyme solution. The red box represents the target protein.

XylA-expressing *E. coli* BL21 cells containing the pET28a-*xylA* plasmid were prepared and selected from kanamycin-resistant plates for verification. As shown in Figure 2A, agarose gel electrophoresis of plasmid DNA isolated from the pET28a-*xylA*-containing clones revealed a specific band at approximately 2700 bp, which was consistent with the target gene fragment of XylA. After culturing the pET28a-*xylA*-BL21 strain and inducing XylA expression, the bacterial cells were collected via centrifugation and subsequently disrupted to obtain a crude enzyme solution containing XylA, followed by protein purification. SDS-PAGE results (Figure 2B) demonstrated that XylA was highly expressed in a soluble form in *E. coli*. A single band was obtained after a series of purification steps, and impure proteins effectively removed. The molecular weight of the target protein was approximately 100 kDa. The concentration of the purified XylA, as determined using the Bradford method, was 0.36 ± 0.02 mg/mL, and its specific enzyme activity was 3.08 ± 0.24 U/mg.

### 2.2. Structural Characterization of XG with Different Fine Structures

XG samples with different fine structures were prepared via enzymatic and chemical treatments. The pyruvate content, acetyl content, monosaccharide composition, and molecular weight of the different samples were analyzed using HPLC, and the specific data presented in Table 1. The results indicated that the pyruvate content gradually decreased from 5.80 ± 0.42% with extension of the enzymatic hydrolysis time. When XylA was used for enzymatic hydrolysis for 5, 15, and 35 min, the pyruvate-group contents of XG-HPM, XG-MPM, and XG-LPM reached 4.81 ± 0.13%, 3.15 ± 0.11%, and 1.05 ± 0.11%, respectively, demonstrating that pyruvated mannose was removed during enzymatic hydrolysis. The acetyl-group contents of XG, XG-HPM, XG-MPM, and XG-LPM were 3.97 ± 0.38%, 4.03 ± 0.11%, 4.03 ± 0.14%, and 4.13 ± 0.27%, respectively, with no significant differences observed. As the enzymatic hydrolysis time increased, the glucuronic acid content gradually decreased, with the contents of XG-HPM, XG-MPM, and XG-LPM being 2.82 ± 0.69%, 1.41 ± 0.42%, and 1.00 ± 0.29%, respectively. Ruijssenaars’ research [16] revealed that XylA converts glucuronic acid into a 4,5-ene-glucuronyl residue. In acidic polysaccharides, the presence of glucuronic acid introduces negative charges into the polysaccharide structure, enabling it to form a three-dimensional gel structure through ionic interactions and thereby enhancing its gel properties [17,18]. However, relatively few studies have reported on the effects that glucuronic acid has on the properties and structure of XG. Therefore, the oxalic acid and dilute alkali treatment groups were established for comparison to investigate the influence that different fine structures have of XG and its properties. The pyruvate and acetyl contents of different treated samples were specifically processed: the pyruvate contents of XG-HPM, XG-MPM, and XG-LPM were comparable to those of XG-HP, XG-MP, and XG-LP, respectively, whereas the acetyl contents of XG-HP, XG-MP, and XG-LP were equivalent to those of XG-A1, XG-A2, and XG-A3, respectively.

The pyruvate contents of the oxalic acid-treated samples (XG-HP, XG-MP, and XG-LP) decreased to 4.92 ± 0.17%, 3.85 ± 0.02%, and 1.42 ± 0.01%, respectively. With an increase in treatment temperature, the pyruvate groups were removed to varying degrees, and the acetyl groups also decreased to some extent, being 3.17 ± 0.14%, 2.98 ± 0.08%, and 2.51 ± 0.12%, respectively. When the pH of the system was <3, both the acetyl and pyruvate groups were removed, which is consistent with the findings of Bradshaw [19]. In XG, pyruvate is linked via an acetal linkage to the O-4 and O-6 positions of terminal mannose residues, while acetyl groups are attached via ester linkages to the O-6 position of internal mannose residues. Both of these linkages are susceptible to acid-catalyzed hydrolysis, and elevated temperature further accelerates this hydrolytic cleavage [20]. When the pH of the system exceeded 9.0, the acetyl groups of XG were gradually removed [21]. Therefore, the dilute alkali method was adopted to prepare samples with different acetyl-group contents. When the KOH concentrations were 1, 2, and 3 mM, the acetyl contents of the samples were 3.38 ± 0.08%, 3.08 ± 0.13%, and 2.58 ± 0.12%, respectively, whereas the pyruvate contents showed no significant differences.

The GPC results for the different XG samples are listed in Table 1. The control group (XG) had the highest molecular weight of 2.2 × 10^7^. In contrast, molecular weights of the enzymatically treated, pyruvate-removed, and acetyl-removed groups showed a decreasing trend. For the enzymatically treated group, the molecular weight decreased from 2.07 × 10^7^ (XG-HPM) to 1.59 × 10^7^ (XG-LPM), indicating that XG lyase specifically and successfully cleaved the side-chain mannose. For the pyruvate-removed group, the molecular weight decreased from 1.17 × 10^7^ (XG-HP) to 0.84 × 10^7^ (XG-LP); for the acetyl-removed group, it decreased from 1.27 × 10^7^ (XG-A1) to 0.96 × 10^7^ (XG-A3). The pyruvate-removed group exhibited an obvious decreasing trend with a larger decline in amplitude than that observed in the dilute alkali treatment group, which may be due to destruction of the main-chain glycosidic bonds caused by high-temperature heating during the chemical pyruvate removal process [22].

The molar ratios of mannose, glucose, and glucuronic acid in the samples varied after different degrees of treatment. The mannose, glucose, and glucuronic acid contents of XG were 52.46 ± 0.91%, 38.74 ± 2.11%, and 8.80 ± 1.35%, respectively. Kang et al. reported that the molar ratios of glucose, mannose, and glucuronic acid in XG were 56.65 ± 1.87%, 39.89 ± 1.41%, and 3.47 ± 0.46%, respectively, which are similar to the results obtained in the present study [23]. With an increase in the degree of enzymatic hydrolysis, the molar ratio of glucose remained relatively stable, whereas the content of mannose in the side chain decreased to 40.42%, 38.54%, and 34.90%, respectively, and the molar ratio of glucose to mannose approached 2:1. The decrease in glucuronic acid content was attributable to the β-elimination reaction of XG lyase, which converted glucuronic acid into a 4,5-ene-glucuronyl residue. For the other samples, molar ratios of the monosaccharides remained stable.

**Figure 3:**
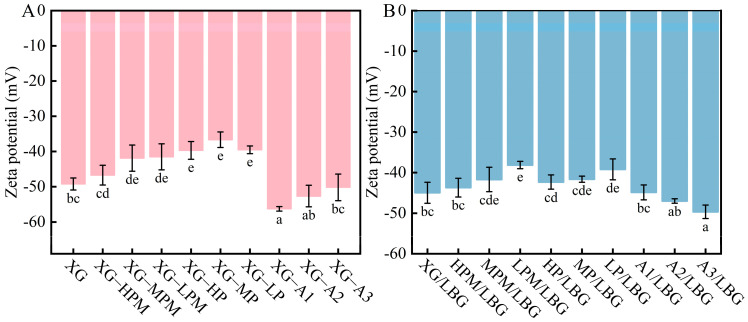
Zeta potential of different XG samples (**A**) and the mixed solution with LBG (**B**). In the sample codes, “XG” stands for commercial xanthan gum as the control group. “HPM”, “MPM”, and “LPM” respectively represent high, medium, and low levels of pyruvated mannan content in the side chains of xanthan gum, while “HP”, “MP”, and “LP” respectively represent high, medium, and low levels of pyruvic acid content. “A1”, “A2”, and “A3” respectively represent low, moderately low, and moderate degrees of acetyl group removal. “LBG” indicates that the XG sample is mixed with LBG. Different lowercase letters indicate significant difference (*p* < 0.05).

The viscosities of XG and its derivatives are shown in Table 1, showing significant differences among the samples of each group. The viscosity of untreated XG was 1654 ± 21 cP. With the extension of enzymatic hydrolysis time (from XG-HPM to XG-LPM), the viscosity significantly increased, ranging from 1713 to 2390 cP. This may be due to the action of XG lyase: the cleavage of side chains reduces steric hindrance, leading to enhanced extensibility of the molecular chains. Xu et al. [24] indicated that different substituents on microbial polysaccharides can maintain or even increase viscosity by enhancing intermolecular interactions when the molecular weight decreases. Figure 3 shows that the ZP of the enzymatic hydrolysis group was slightly lower than that of the pyruvate-removed group, indicating that the exposure of glucuronic acid groups slightly enhanced the negative charge, whereas the retention of acetyl groups maintained the hydrophobic interaction of molecular chains and increased the internal friction of the solution, thus increasing viscosity. Viscosities of samples from the pyruvate-removed group were low and relatively similar, with no significant differences, being 1381 ± 11, 1430 ± 26, and 1418 ± 7 cP, respectively. Viscosities of samples from the acetyl-removed group were also relatively low (1499 ± 24, 1480 ± 3, and 1479 ± 10 cP, respectively). Carboxyl groups in the pyruvate groups can form hydrogen bonds, and simultaneously, these are partially ionized in water. Negative charges generate repulsive forces between XG molecular chains, and their absence directly weakens intermolecular forces, thereby reducing the entanglement density and network structural strength of the XG solution [25]. Acetyl groups are present in the mannose units of XG that are connected to the main chain, which are crucial for maintaining stability of the double-helix structure. The hydrolysis of acetyl groups causes dissociation of the double-helix structure, weakens the rigidity of XG molecules, and decreases viscosity [26]. In addition, cleavage of the chain and the decrease in molecular weight during treatment are potential reasons for the reduction in viscosity observed [27].

### 2.3. FTIR and XRD Analysis

To investigate the effects of different treatment approaches on the structure and conformation of XG, FTIR and XRD analyses were performed on native XG and three representative derivatives—XG-LPM, XG-LP, and XG-A3. These samples were selected because they represent the highest degree of modification within each treatment strategy (enzymatic hydrolysis, oxalic acid treatment, and dilute alkali treatment, respectively), which allows the clearest spectroscopic detection of structural and conformational changes relative to the native control.

**Figure 4:**
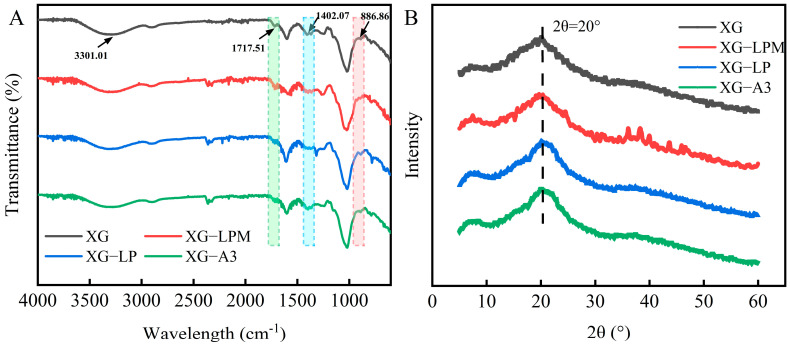
FT−IR spectra (**A**) and XRD spectra (**B**) of XG, XG−LPM, XG−LP and XG−A3.

FTIR spectroscopy serves as a qualitative tool for probing alterations in molecular configurations and functional groups. As illustrated in Figure 4A, all normalized spectra exhibited characteristic peaks corresponding to their respective components. Previous studies have identified that absorption at approximately 3300 cm^−1^ arises from O-H stretching vibrations, whereas the peak near 1402 cm^−1^ corresponds to bending modes of carboxyl groups. Additionally, acetyl-related signals typically emerge within the range of 1600–1700 cm^−1^, whereas a distinct peak at approximately 886 cm^−1^ indicates the presence of mannose residues [28,29]. Disappearance of the peak at 886 cm^−1^ in the FTIR spectrum of XG-LPM validates that pyruvated mannose units on the side chains of XG were removed after enzymatic hydrolysis. The reduced intensity of the 1402 cm^−1^ band in both XG-LPM and XG-LP suggests a diminished carboxyl content during elimination of the pyruvate group. Similarly, the decreased absorbance at 1717 cm^−1^ for XG-LP and XG-A3 reflects partial deacetylation induced by specific treatments. Overall, no additional spectral deviations distinguished the modified forms from untreated XG, implying that the main structure of the polysaccharide was preserved despite undergoing chemical or enzymatic modifications [5].

XRD is commonly used to characterize the crystal structures of polysaccharides. As shown in Figure 4B, all samples exhibited a dominant diffraction peak, indicating that XG treated with different methods predominantly exists in the form of semi-crystalline and amorphous states [5]. Differences were observed in the position and intensity of the diffraction peaks among the various samples, with a higher peak intensity corresponding to a more disordered conformation of the sample [30]. XG-A3 exhibited the highest peak intensity at approximately 2θ = 20°, whereas XG and XG-LPM presented similar peak intensities. Thus, the conformation of XG may have undergone changes after deacetylation. These results demonstrate that different treatment methods affect the conformation of XG without altering its main structure.

### 2.4. Zeta Potential

ZP values of the different modified XG samples are shown in Figure 3. All samples exhibited negative ZP values, indicating that they are anionic polysaccharides. The ZP of the XG control group was −47.13 ± 3.96 mV, which is attributable to carboxyl groups carried by the pyruvate and glucuronic acid groups. These carboxyl groups ionize into negatively charged carboxylate anions in water, directly increasing the net negative charge density on the surface of XG molecules.

ZP values of the enzymatic hydrolysis (XG-HPM, XG-MPM, and XG-LPM) and pyruvate-removed (XG-HP, XG-MP, and XG-LP) groups all increased, being −46.73 ± 2.80, −41.9 ± 4.69, −41.53 ± 3.70, −39.7 ± 2.52, −35.23 ± 6.85, and −44.07 ± 3.75 mV, respectively. This was due to removal of the pyruvate group during treatment, resulting in a reduction in the negative charge. The ZP values of XG-HP and XG-MP were slightly higher than those of the enzymatic hydrolysis group, whereas that of XG-LP was lower, showing an overall trend of initially increasing and then decreasing. This may be because acetyl groups in the pyruvate-removed group were removed to a certain extent, leading to a decrease in order of the XG molecular structure. This could cause a certain degree of collapse or aggregation of the molecular chains, making it more difficult for some negatively charged groups inside the molecules to be approached by ions in the solution, thus reducing the effective negative charge.

The acetyl-removed groups (XG-A1, XG-A2, and XG-A3) exhibited the lowest ZP values, which were −56.23 ± 0.64, −52.63 ± 5.02, and −50.17 ± 4.75 mV, respectively. This may be due to two reasons: on the one hand, the retention of pyruvate groups maintained the number of negative charges; on the other hand, the removal of acetyl groups loosened the double-helix structure of XG, thus exposing the negatively charged groups.

The ZP values of mixed solutions of the enzymatic hydrolysis and pyruvate-removed groups were higher than those of the control group, whereas ZP values of the mixed solution of the acetyl-removed group were lower than those of the control group. This trend is consistent with the ZP-value trend of the different modified XG solutions. ZP values of the mixed solutions were generally higher than those of the corresponding XG solutions because LBG is a neutral polysaccharide composed of mannose and galactose units with a small number of negative charges [14]. This was consistent with the conclusions of previous ZP studies on LBG in other blending systems [31]. As a non-ionic polysaccharide, LBG molecules contain abundant hydroxyl groups but lack carboxyl or sulfate groups, and therefore carry only trace negative charges. Thus, the addition of LBG does not introduce significant additional charge to the mixed system.

### 2.5. Texture Analysis

**Figure 5:**
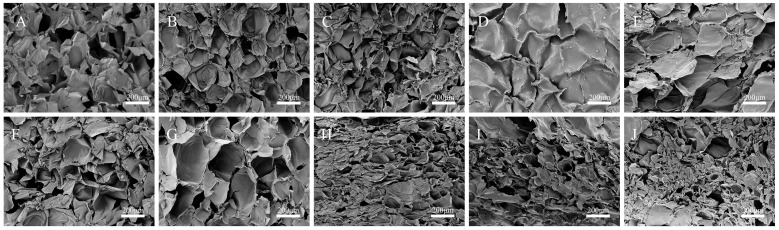
SEM images of different composite gels. (**A**) XG/LBG, (**B**) HPM/LBG, (**C**) MPM/LBG, (**D**) LPM/LBG, (**E**) HP/LBG, (**F**) MP/LBG, (**G**) LP/LBG, (**H**) A1/LBG, (**I**) A2/LBG, (**J**) A3/LBG.

Gel texture characteristics of the different samples are listed in Table 2. All the composite gels prepared with native and modified xanthan gum exhibited homogeneous appearance, and no obvious difference in visual appearance was observed among different samples. For the enzymatically hydrolyzed groups (HPM/LBG, MPM/LBG, and LPM/LBG), an increase in enzymatic hydrolysis time significantly decreased hardness, adhesiveness, and chewiness compared with those of the control (XG/LBG). This decreasing trend may be attributable to the unsubstituted galactomannan regions of LBG, which can form hydrogen bonds with XG chains and generate hydrophobic microdomains [32]. As the hydrolysis time increases, a higher proportion of mannose at the end of the side chains would be cleaved, leading to shortened XG side chains and reduced binding sites for LBG, which significantly decreases the crosslinking density of the three-dimensional network. Consistent with the scanning electron microscopy (SEM) results (Figure 5B–D), the dense network became loose and porous as the degree of enzymatic hydrolysis increased. Elasticity refers to the ability of a sample to recover its original shape after being subjected to pressure [33]. LPM/LBG exhibited the lowest elasticity, showing a significant difference from other samples. This may be due to the reduced gel-forming ability caused by high enzymatic hydrolysis degree, approaching the gelation critical point [16]. Cohesiveness reflects the consistency and stability of the internal structure of a gel. After enzymatic cleavage of the terminal mannose residue, cohesiveness increased, possibly because the shorter and more regular side chains reduced steric hindrance, improved the efficiency of hydrogen-bond formation between the XG side chains and LBG, and ultimately enhanced the internal bonding of the gel. Glucuronic acid contributes to the formation of dense gels between XG and LBG through hydrogen bonding and electrostatic interactions. With an increase in enzymatic hydrolysis time, the glucuronic acid contents of XG-HPM, XG-MPM, and XG-LPM decreased to 2.82 ± 0.69%, 1.41 ± 0.42%, and 1.00 ± 0.29%, respectively. Hardness, chewiness, adhesiveness, and elasticity are closely related to the strength of a three-dimensional gel network. Wang et al. [34] demonstrated that ordered xanthan interacts with LBG through side chains driven by electrostatic interactions, while disordered xanthan binds LBG via backbone interactions dominated by hydrogen bonds. Glucuronic acid enhances these interactions by providing additional electrostatic attraction and hydrogen-bonding sites. The conversion of glucuronic acid to a 4,5-ene-glucuronyl residue may weaken the interactions with LBG chains.

In the pyruvate-removed group, hardness significantly decreased, cohesiveness slightly increased, elasticity barely changed, and adhesiveness and chewiness fluctuated to a certain extent. The reduction in pyruvate groups led to fewer polar sites for intermolecular binding and decreased crosslinking density, which partially caused a change in hardness. The removal of pyruvate groups reduced electrostatic repulsion and improved stability of the XG helical structure [35], which may be the reason for the increased gel cohesiveness observed. Adhesiveness is defined as the product of hardness and cohesiveness, and it reflects the ability of food components to adhere to each other and resist separation during stress. Both the enzymatic hydrolysis (HPM/LBG, MPM/LBG, and LPM/LBG) and pyruvate-removed (HP/LBG, MP/LBG, and LP/LBG) groups showed a decreasing trend in adhesiveness, with the enzymatic hydrolysis group exhibiting the most significant decrease. The hardness of these gels also showed a synchronous decreasing trend, indicating that loosening of the three-dimensional network structure synchronously affected gel adhesiveness. Compared with the pyruvate-removed group, the enzymatic hydrolysis group had similar pyruvate contents, and the differences originated from the shorter side chains and glucuronic acid and acetyl contents. Enzymatic hydrolysis cleaved the terminal mannose residues present in the side chains, leading to a further reduction in crosslinking sites and density between XG and LBG, thereby resulting in further decreases in hardness, adhesiveness, and chewiness. In addition, the introduction of double bonds through glucuronic acid conversion weakened the flexibility of the side chains, which may be the reason for the decreased elasticity and increased cohesiveness observed [36].

In the acetyl-removed groups (A1/LBG, A2/LBG, and A3/LBG), the changes in cohesiveness, elasticity, adhesiveness, and chewiness were small, with no significant differences, indicating that these properties are less affected by acetyl-mediated interactions. The gel hardness showed a trend of initially decreasing and then increasing. This may be because the reduction in acetyl content weakened the hydrophobic interactions, loosened the XG double-helical structure, and decreased molecular rigidity. When the double-helical structure loosened to a certain extent, the side chains were fully exposed, enhancing hydrogen bonding between the molecular chains and forming a denser network structure. As observed in the SEM images (Figure 5H–J), the gel network became denser and more porous with the removal of acetyl groups. Compared with the acetyl-removed group, the pyruvate-removed group had a similar acetyl content, and differences originated from the pyruvate content. The presence of more pyruvate can provide more binding sites, resulting in a higher crosslinking density between the XG side and LBG chains. Therefore, the acetyl-removed group generally had higher hardness, adhesiveness, and chewiness than the pyruvate-removed group did. Fitzpatrick et al. [37] investigated XG-konjac glucomannan (KGM) mixed gels and reported that deacetylated XG/KGM gels exhibited stronger gel strength than native and depyruvated samples. This ranking is consistent with our findings.

In this study, the relationship between polysaccharide molecular weight and gel textural properties was consistent with the results of previous studies. Rodríguez-Dorado et al. [38] pointed out that the molecular weight of polysaccharides has a significant impact on the texture of gels, and Zhang et al. [39] found that low-molecular-weight yeast β-glucan made composite gels softer. Thie present study also observed that molecular weight was negatively correlated with gel hardness, chewiness, adhesiveness, and elasticity while being positively correlated with cohesiveness. The XG control group had the highest molecular weight, which was significantly different from that of the other samples. However, the hardness of XG/LBG was lower than that of the acetyl-removed group. The pyruvate- and acetyl-removed groups had similar molecular weights; however, significant differences in hardness, adhesiveness, and chewiness were noted, with small differences observed in other indicators. This indicates that, within the scope of this study, the regulatory effect of fine structural changes on gel characteristics is more significant than that of molecular weight degradation and the impact of molecular weight.

### 2.6. Rheological Properties

**Figure 6:**
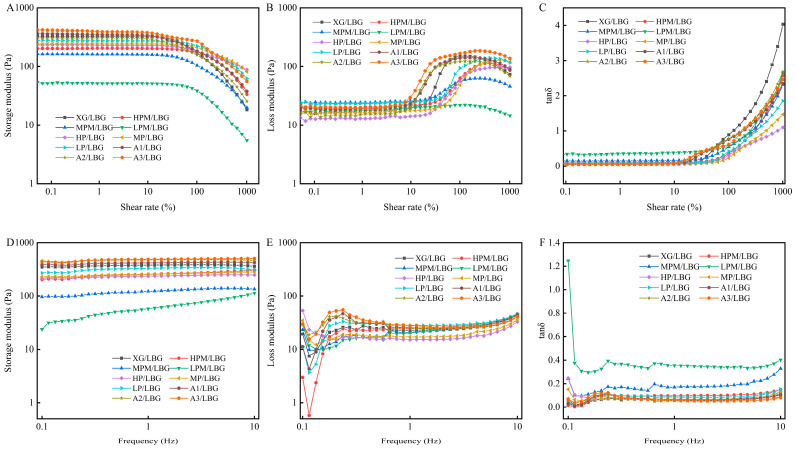
Amplitude sweep curves (**A**–**C**) and frequency sweep curves (**D**–**F**) of different composite gels. In the sample codes, “XG” stands for commercial xanthan gum as the control group. “HPM”, “MPM”, and “LPM” respectively represent high, medium, and low levels of pyruvated mannan content in the side chains of xanthan gum, while “HP”, “MP”, and “LP” respectively represent high, medium, and low levels of pyruvic acid content. “A1”, “A2”, and “A3” respectively represent low, moderately low, and moderate degrees of acetyl group removal. “LBG” indicates that the XG sample is mixed with LBG.

Amplitude sweep tests describe the characteristics of samples within a nondestructive deformation range by varying the strain amplitude at a fixed frequency and temperature. The amplitude sweep results for the different gel samples are shown in Figure 6A–C. Within the low shear strain range (shear strain < 10%), all gel samples exhibited linear viscoelastic responses; the *G′* and *G″* remained constant, with *G′* > *G″*. The *tanδ* was maintained at a low level, indicating that the three-dimensional elastic network structure of the gel was stable and the mechanical behavior dominated by elasticity. As the shear strain increased, the gel network was destroyed, *G′* significantly decreased, and *G″* increased; *tanδ* changed from <1 to >1, indicating that the gel transformed from an elasticity-dominated state to a viscosity-dominated state, undergoing a yield phenomenon [40]. Liu et al. [5] investigated the rheological behaviors of synergistic gels composed of xanthan and galactomannan. Their rheological results showed that deacetylation significantly increased the *G′* and gel strength, while depyruvylation decreased *G′* and weakened the network. This tendency was consistent with our present study, in which the removal of side-chain substituents promoted the disorder–order conformational transition of xanthan and further regulated the rheological properties of the synergistic system.

Frequency scanning is typically used to detect the viscoelastic behavior of gels within the LVR. The frequency sweep results for the different gel samples are shown in Figure 6 D–F. Within the test frequency range, the *G′* of all gel samples was consistently higher than the *G″*, and *tanδ* was much lower than 1, indicating that the mechanical behavior of gels was dominated by elasticity, and the three-dimensional elastic network structure was stable [41]. Taking the XG/LBG synergistic gel as the control, *G′* values of composite gels from the acetyl-removed groups (A1/LBG, A2/LBG, and A3/LBG) were higher than that of the control. Moreover, the *G′* was stable in the low-frequency region and only slightly increased in the high-frequency region. Acetyl groups hinder hydrogen bonding between the XG side chain and the exposed regions of the LBG main chain through steric hindrance. Removing acetyl groups can reduce the steric hindrance effect, fully expose the side chain, and enhance the hydrogen bond crosslinking density. The *G′* values of the pyruvate-removed groups (HP/LBG, MP/LBG, and LP/LBG) were lower than that of the control, which was attributable to the removal of pyruvate groups, leading to weakened local electrostatic interactions and reduced polar sites for intermolecular binding, thus decreasing the gel network strength. The *G′* values of composite gels from the enzymatic hydrolysis groups (HPM/LBG, MPM/LBG, and LPM/LBG) were significantly lower than those of other groups, and their *tanδ* values were relatively high. This may be because enzymatic degradation removed the pyruvated mannose residues, further reducing the number of polar sites available for intermolecular binding. Meanwhile, the conversion of glucuronic acid affected local electrostatic interactions, resulting in weakened polar interactions. These findings are consistent with the previous gel hardness analysis results.

**Figure 7:**
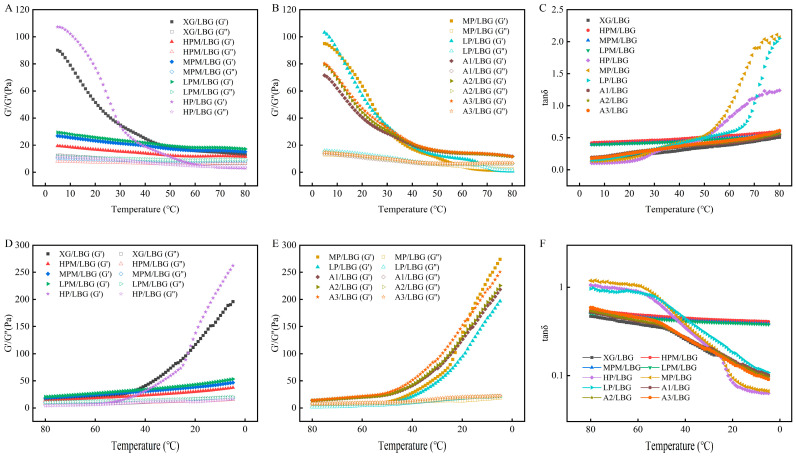
Temperature sweep curves of the heating process (**A**–**C**) and cooling process (**D**–**F**) of different mixed solutions. In the sample codes, “XG” stands for commercial xanthan gum as the control group. “HPM”, “MPM”, and “LPM” respectively represent high, medium, and low levels of pyruvated mannan content in the side chains of xanthan gum, while “HP”, “MP”, and “LP” respectively represent high, medium, and low levels of pyruvic acid content. “A1”, “A2”, and “A3” respectively represent low, moderately low, and moderate degrees of acetyl group removal. “LBG” indicates that the XG sample is mixed with LBG.

Figure 7A–F illustrates the changes in *G′*, *G″*, and *tanδ* of different XG/LBG mixed solutions during heating and cooling from 5 °C to 80 °C. As shown in Figure 7A–C, during heating, the *G′* and *G″* values of all samples decreased with increasing temperature. Heating disrupted the intermolecular hydrogen bonds, and viscoelasticity transformed from an elasticity-dominated to viscosity-dominated state. With an increase in temperature, the *tanδ* of all samples slowly increased. When the temperature exceeded 40 °C, the increase rate of *tanδ* accelerated and tended to stabilize after exceeding 60 °C. Except for HP/LBG, MP/LBG, and LP/LBG, no intersections between the *G′* and *G″* curves of the other mixed solution samples were observed, and *tanδ* was always <1. This indicates that the contribution of viscosity gradually increased relative to the elasticity but did not completely dominate. The *tanδ* values of HP/LBG, MP/LBG, and LP/LBG continued to increase with temperature and exceeded 1, showing valleys at 80 °C. This indicated that the increase in temperature transformed the mixed system from an elasticity-dominated network structure to a viscosity-dominated fluid-like state, similar to the results of Kim et al. [42]. Liu et al. [5] showed that the reduction in pyruvate content increased the order-disorder conformational transition temperature of XG, inhibited the synergistic effect between XG and *G. sinensis* gum, and rapidly decreased the *G′* and *G″* values with increasing temperature, showing valleys at approximately 80 °C. The removal of acetyl groups led to an increase in the conformational disorder of XG, further improving extensibility and flexibility of the molecular chains and enhancing intermolecular interactions. As shown in Figure 7D–F, during the cooling process, the *G′* and *G″* significantly and synchronously increased with a decrease in temperature. During cooling, the thermal motion of molecules weakened, the mechanical behavior transformed from a viscosity-dominated state back to an elasticity-dominated state, and the process was thermally reversible. The *tanδ* of the mixed system significantly decreased with temperature reduction and was <1, indicating that elasticity re-dominated the mechanical behavior, reflecting the reconstruction ability and thermal reversibility of the gel network [43]. Differences in the curves of various mixed systems reflect the differences in their synergistic gelation abilities. During cooling, as the temperature decreased, the *G′* and *G″* of HPM/LBG, MPM/LBG, and LPM/LBG did not sharply increase, and the curves were gentle, indicating a decrease in the ability to form a three-dimensional network structure through crosslinking.

### 2.7. Moisture Distribution Analysis

**Figure 8:**
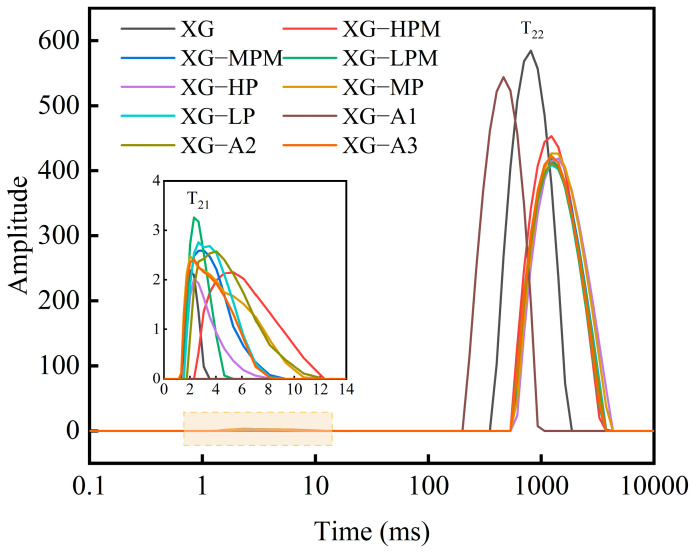
LF−NMR curves of different gel samples. In the sample codes, “XG” stands for commercial xanthan gum as the control group. “HPM”, “MPM”, and “LPM” respectively represent high, medium, and low levels of pyruvated mannan content in the side chains of xanthan gum, while “HP”, “MP”, and “LP” respectively represent high, medium, and low levels of pyruvic acid content. “A1”, “A2”, and “A3” respectively represent low, moderately low, and moderate degrees of acetyl group removal. The inset is a magnified view of the orange area.

LF-NMR technology uses the spin–spin relaxation phenomenon of hydrogen protons in a low magnetic field to indirectly reflect the state of water molecules. It records the T_2_ and corresponding proportions to investigate the state, migration, and distribution of moisture in samples. A smaller T_2_ value indicates tighter binding between water molecules and nonaqueous components. The T_2_ can represent several different states of moisture based on its duration: specifically, T_21_ (0~10 ms) is bound water; T_22_ (10~100 ms) is immobilized water; and T_23_ (>100 ms) is free water [44]. The moisture distribution results for different gel samples are shown in Figure 8. All samples exhibited two peaks (T_21_ and T_23_), with the T_23_ peak having the largest area. This indicates that all gel samples contained a small amount of bound water and large amount of free water, and that free water was the main form of moisture present in all gel samples. For the gels formed using modified XG and LBG, the peak vertex time shifted toward longer relaxation times, suggesting enhanced freedom of molecular motion and reduced local environmental constraints, which may be attributable to the decrease in molecular weight observed. As shown in Figure 5 the other gels formed by modified XG and LBG exhibited a thinned network structure and increased pores, which may also be a reason for the shifts in peak vertex time toward longer relaxation times. The T_23_ relaxation time of the deacetylated groups progressively increased with increasing degree of deacetylation. Zeng et al. [45] demonstrated that deacetylated konjac glucomannan facilitates the transformation of semi-bound water into free water within the gel network. Similarly, a water migration trend comparable to that of the deacetylated groups was observed in the gel as the pyruvate content decreased, indicating enhanced water mobility and a compromised gel structure. Liu et al. [25] reported that the removal of pyruvate groups reduced the charge density of XG, thereby attenuating its interaction with proteins.

### 2.8. Thermal Stability

**Figure 9:**
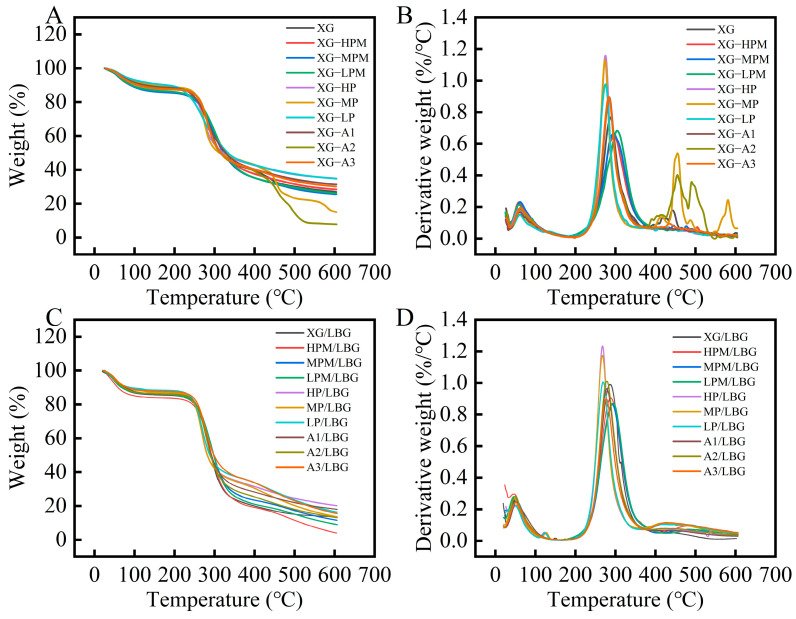
(**A**) TG and (**B**) DTG curves of different modified xanthan gum samples without LBG and (**C**) TG and (**D**) DTG curves of different composite gels. “HPM”, “MPM”, and “LPM” respectively represent high, medium, and low levels of pyruvated mannan content in the side chains of xanthan gum, while “HP”, “MP”, and “LP” respectively represent high, medium, and low levels of pyruvic acid content. “A1”, “A2”, and “A3” respectively represent low, moderately low, and moderate degrees of acetyl group removal.

TG curves describe the weight loss of samples, whereas derivative TG (DTG) curves reflect the weight-loss rate. The TG and DTG curves of different modified samples are shown in Figure 9A,B, all modified XG samples exhibited a similar three-stage weight-loss profile. The enzymatic hydrolysis group (XG-HPM, XG-MPM, XG-LPM) showed progressively lower weight-loss rates and higher maximum decomposition temperatures with increasing hydrolysis time. The pyruvate-removed group (XG-HP, XG-MP, XG-LP) exhibited the lowest weight-loss rates among all samples. The acetyl-removed group (XG-A1, XG-A2, XG-A3) showed intermediate thermal stability. To specifically assess the thermal stability of the composite gel network, TG analysis was conducted on lyophilized composite gels. The TG curves of all gel samples are shown in Figure 9C. All gels exhibited three distinct weight-loss stages. The first weight loss of samples occurred in the temperature range of 25–150 °C, which was mainly attributable to the evaporation of free and bound water in the samples. Furthermore, the double weight-loss peak observed at this stage (Figure 9D) may have been caused by different evaporation temperatures resulting from the varying binding capabilities of water in the gel. The second stage of significant weight loss occurred between 200 °C and 400 °C, primarily due to the destruction of gel structures and molecular chains. The order of mass loss for the different gel samples in this stage was as follows: LPM/LBG (66.48%) > XG/LBG (65.64%) > MPM/LBG (63.65%) > HPM/LBG (63.34%) > A2/LBG (59.46%) > A1/LBG (56.86%) > HP/LBG (53.44%) > MP/LBG (53.34%) > A3/LBG (51.74%) > LP/LBG (50.37%). Results indicated that the different modified XG samples exerted varying improvements in thermal stability of the gels. Among them, the pyruvate-removed group showed the lowest weight-loss rate, whereas the acetyl-removed group exhibited a weight-loss rate between that of the control and pyruvate-free groups. This may be because the removal of pyruvate groups increased the ordered conformation of XG, which affects its thermal stability [5]. In the enzymatic hydrolysis group, the weight-loss rates of HPM/LBG and MPM/LBG were slightly lower than those in the control group, whereas the weight-loss rate of LPM/LBG was higher. This could be ascribed to moderate enzymatic treatment that partially removed pyruvate to enhance the ordered conformation and improve thermal stability of the composite gels; however, excessive treatment (LPM) might reduce gel synergism, leading to an increased weight-loss rate. The third weight-loss stage occurred from 400–600 °C, where small molecules continued to carbonize to form simple compounds [46].

The DTG curves of different gel samples are shown in Figure 9D. The order of maximum weight-loss temperature for different gels were observed as follows: LPM/LBG (294.32 °C) > MPM/LBG (291.94 °C) > HPM/LBG (288.29 °C) > XG/LBG (286.84 °C) > A1/LBG (278.62 °C) > A2/LBG (278.59 °C) > A3/LBG (277.01 °C) > LP/LBG (269.14 °C) > MP/LBG (267.79 °C) > HP/LBG (267.47 °C). Enzymatic treatment increased the maximum weight-loss temperature of the composite gels, which was consistent with the findings reported by Sousa et al. [47]. Sousa et al. demonstrated that enzymatic treatment shifted the main degradation stage of the polysaccharide network to a higher temperature range, which could be attributed to the removal of carboxyl groups by enzymatic cleavage, leading to a reduction in bound water and thus enhanced structural resistance. XG can form hydrogen bonds with unsubstituted regions in the main chain of LBG via hydroxyl and carboxyl groups on its side chains, thereby enabling tight crosslinking between the two polysaccharides to form a dense and stable initial gel network. Meanwhile, contents of the acetyl and pyruvate groups affect the ordered conformational changes in XG, indirectly influencing their interactions and playing a crucial role in the stability of composite gels.

### 2.9. Microstructure

Figure 5 shows SEM images of the freeze-dried composite gels. Pore indices of different gel samples are presented in Table 3. The XG/LBG composite gel exhibited a porous network structure with a relatively thick and uniformly sized framework (Figure 5A), which serves as a core structure for supporting the gel morphology. This dense and homogeneous coarse-framework network corresponds to the short T_2_ observed in the LF-NMR analysis, indicating that water molecules are tightly confined within the interchain spaces with restricted molecular chain mobility. Consequently, this structural integrity manifested as a relatively high *G′* in the rheological measurements, underscoring the pivotal role of the stable network in reinforcing macroscale mechanical properties of the gel.

Figure 5B–D presents the composite gels formed through enzyme-treated XG and LBG. Compared with XG/LBG, the originally dense and thick bundled framework became thinner, the porosity increased, and the gel network turned porous and uniform. Such structural alterations are associated with a slight prolongation in the T_2_, modest increase in the proportion of free water, and moderately enhanced molecular mobility. With an increase in the degree of enzymatic treatment, the pores became smaller and irregularly distributed. The gel surface of LPM/LBG became smooth and sheet-like, and the densely intertwined thick bundled network disappeared, which was replaced by many scattered, small segments. After specific degradation with XylA, the binding sites between XG and LBG decreased, leading to a reduced synergistic interaction. At this point, the gel crosslinking density was substantially diminished, accompanied by a further prolongation in the T_2_, marked increase in the proportion of free water, and continuous decline in rheological performance—exhibiting a *G′* significantly lower than that of the other samples.

Figure 5E–G displays the microtopography of the freeze-dried surfaces of HP/LBG, MP/LBG, and LP/LBG. Pyruvate groups can promote the stability of the XG helical conformation via negative charge-mediated electrostatic interactions and polar hydrogen bonding, while their polar sites can enhance the synergistic interactions with LBG [37]. With the removal of pyruvate, the porosity increased, porous network became loose and inhomogeneous, with a mixture of large and small pores, and the framework became thinner. These loose and heterogeneous structural characteristics correlate with the prolongation in the T_2_, increased proportion of free water, and markedly enhanced molecular chain mobility. Macroscopically, this translates to moderate rheological properties, being weaker than those of the control group owing to the loose network structure yet superior to those of the enzymatically hydrolyzed groups as a result of less pronounced changes in heterogeneity and molecular mobility.

Figure 5H–J shows the microtopography of the freeze-dried surfaces of A1/LBG, A2/LBG, and A3/LBG. After the removal of acetyl groups, SEM images of the composite gels exhibited a denser network and smaller, uniformly distributed pores. This may be attributable to the removal of acetyl groups, which increased the conformational disorder of XG, reduced intramolecular hydrogen bonds, and rendered the molecular chains longer and more flexible [5]. Meanwhile, the removal of acetyl groups reduced steric hindrance and promoted synergistic interactions between XG and LBG, which may explain the improved hardness, elasticity, and stability of the gels observed. Such microstructural advantages directly translate into improved macroscale rheological properties, such as enhanced gel hardness, elasticity, and stability. This observation forms a closed-loop validation with the higher *G′* values obtained from rheological measurements.

### 2.10. Correlation Analysis

**Figure 10:**
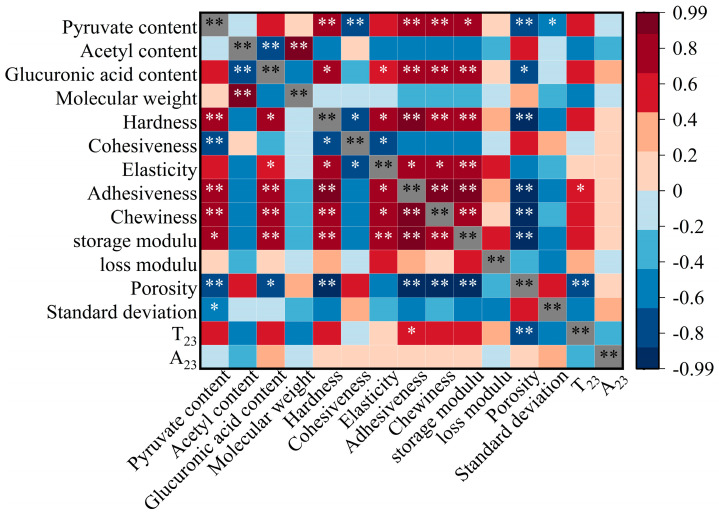
Correlation analysis between fine structure and texture characteristic indicators of XG. * indicates significant correlation (*p*−value < 0.05), ** indicates extremely significant correlation (*p*−value < 0.01).

To further investigate the core mechanism involved and modification regulation rules of the XG/LBG synergistic gelation, this study analyzed correlations among the contents of pyruvate, acetyl, and glucuronic acid in XG and the gel texture, rheology, LF-NMR, and SEM data. As shown in Figure 10, the results showed a strong overall correlation, with 53 positive correlation coefficients, 53 negative correlation coefficients, and 33 coefficients ≥0.5. This correlation analysis provides support for the regulatory effect of XG on gel properties through the “structure–crosslinking–performance” mechanism.

From the perspective of the correlation among groups and properties, pyruvate and glucuronic acid exhibited highly consistent positive regulatory characteristics: both showed significant or extremely significant positive correlations with gel hardness, gumminess, chewiness, and the G′, as well as significant or extremely significant negative correlations with porosity. Additionally, the pyruvate content had a significantly negative correlation with the SD of pore size. These results indicate that the two groups can jointly increase crosslinking node density, network compactness, and uniformity through polar interactions, such as by providing negative charges and forming hydrogen bonds, thereby enhancing gel strength. The similarity in their correlations with texture indicators essentially stems from the synergistic effect of their polar functional groups in network stabilization. In contrast, acetyl groups exhibited a negative regulatory effect, showing extremely significant negative correlations with strength-related indicators, such as hardness and the G′, and a positive correlation with porosity. This confirmed that steric hindrance inhibited hydrogen-bond formation between XG and LBG.

This correlation directly supports the regulatory mechanism of modification; pyruvate, acetyl, and glucuronic acid on the side chain of XG mediate polarity, charge interactions, and steric hindrance to form hydrogen bonds that crosslink with unsubstituted regions of the LBG side chain, thereby constructing a three-dimensional network. Modification differentially alters crosslinking efficiency and network characteristics via targeted regulation of the content of these groups and the molecular structure: pyruvate removal reduces binding sites and crosslinking density, leading to a loose and heterogeneous microscopic gel network and decreased macroscopic strength; enzymatic hydrolysis impairs XG chain integrity, further weakening the crosslinking density; moderate removal of acetyl groups lowers steric hindrance, rendering the XG conformation more disordered and molecular chains more flexible, which significantly improves the efficiency of hydrogen-bond formation with LBG, thereby increasing the crosslinking density. Ultimately, this results in optimized properties, such as reduced microscopic porosity and significantly enhanced macroscopic properties, such as G′ and hardness.

## 3. Materials and Methods

### 3.1. Materials

XG was kindly provided by the Shandong Fufeng Group (Linyi, China) with an initial pyruvate content of 5.80 ± 0.42%, an acetyl content of 3.97 ± 0.38%, a weight-average molecular weight (Mw) of 2.20 × 10^7^ Da, and a monosaccharide composition of glucose, mannose, and glucuronic acid at molar ratios of 52.46 ± 0.91%, 38.74 ± 2.11%, and 8.80 ± 1.35%, respectively (detailed data are provided in Table 1). LBG was purchased from LBG Sicilia Ingredients (Sicily, Italy), the galactose: mannose ratio of LBG was 1:3.78, which was tested according to previous method [48]. Dimethyl silicone oil supplied by Dow Corning Investment Co., Ltd. (Midland, MI, USA). Tryptone and yeast extract were obtained from Oxoid (Basingstoke, UK). Kanamycin was purchased from Takara Biomedical Technology (Beijing) Co., Ltd. (Beijing, China). Imidazole and TRIS were acquired from Amresco (Solon, OH, USA). Competent cells, restriction enzymes, and isopropyl β-D-1-thiogalactopyranoside (IPTG) were procured from Beijing TransGen Biotech Co., Ltd. (Beijing, China). Chromatography-grade acetonitrile was purchased from Fisher Scientific Company LLC (Pittsburgh, PA, USA), and Milli-Q ultrapure water used in the chromatographic experiments. All other chemical reagents were of analytical grade and purchased from Sinopharm Chemical Reagent Co., Ltd. (Shanghai, China).

### 3.2. Heterologous Expression of Xanthan Lyase

#### 3.2.1. Cloning the Xanthan Lyase-Encoding Gene and Constructing Its Expression Vector

The coding sequence of the xanthan lyase (*xylA*) gene (GenBank: AF242413.1) was retrieved from the NCBI database. Synthesis of the recombinant pET28a-*xylA* plasmid for XylA expression was performed by GenScript Biotech Corporation (Nanjing, China). The pET28a-*xylA* plasmid was chemically transformed into *Escherichia coli* BL21, whereafter the cells were spread onto kanamycin-resistant plates and incubated overnight at 37 °C. Positive clones were selected and verified via colony PCR amplification using a Biometra Tone 96G PCR Thermocycler (Analytik Jena AG, Jena, Germany).

#### 3.2.2. Heterologous Expression and Purification of Xanthan Lyase

The XylA-expressing strain, pET28a-*xylA*-BL21, was inoculated into Luria–Bertani (LB) medium containing 50 μg/mL kanamycin and cultured with shaking at 37 °C until an optical density at 600 nm (OD600) of 0.6–0.8 was reached. Protein expression was induced by adding 0.5 mM IPTG at 16 °C with shaking at 200 rpm overnight. Cells were harvested via centrifugation, resuspended in Tris buffer (pH 8.0), and disrupted using a JY98-IIIDN Ultrasonic Cell Disruptor (Ningbo Scientz Biotechnology Co., Ltd., Ningbo, China) under the following conditions: 60% power and working for 4 s at 4 s intervals for a total duration of 10 min. The lysate was centrifuged at 10,000 rpm for 30 min to separate the supernatant from inclusion bodies.

The supernatant was filtered through a 0.45 μm membrane and purified using an NGC Chromatography System Quest 100 Plus (Bio-Rad Laboratories, Hercules, CA, USA) with a Ni-NTA affinity column. After sample loading, the column was washed with elution buffer (50 mM Tris, 500 mM NaCl, and 500 mM imidazole, pH 8.0) using a gradient elution. The target protein peak was collected, and its molecular weight verified via sodium dodecyl sulfate–polyacrylamide gel electrophoresis (SDS-PAGE). Xanthan lyase activity was determined as described by Ruijssenaars [16]. The obtained XG lyase activity was 0.12 ± 0.01 U/mL, with the optimal enzymatic conditions being 40 °C and pH 6.5.

### 3.3. Preparation of XG Samples with Different Fine Structures

A volume of 0.1 U purified XylA solution was added to 1 L XG solution (5 g/L, pH 6.5) and enzymatically hydrolyzed at 40 °C for either 5, 15, or 35 min. The reaction mixture was then placed in an ice bath to terminate the reaction. Thereafter, the XG hydrolysates were precipitated with anhydrous ethanol (1:4, *v*/*v*), dried at 50 °C for 4 h, ground into a powder, and passed through an 80-mesh sieve. Based on the hydrolysis time, the enzymatically hydrolyzed XG samples were named XG-HPM (5 min), XG-MPM (15 min), and XG-LPM (35 min).

A total of 5.0 g XG powder was fully dissolved in 1 L of a solution containing 10 mM H_2_C_2_O_4_ and 0.1 M KCl and then heated at either 65 °C, 80 °C, or 95 °C for 2 h. After cooling to room temperature, the pH of the solution was adjusted to 7.0. The XG derivatives were precipitated with anhydrous ethanol (1:4, *v*/*v*), dried at 50 °C for 4 h, ground into a powder, and passed through an 80-mesh sieve. Based on the reaction temperatures, the obtained samples were named XG-HP (65 °C), XG-MP (80 °C), and XG-LP (95 °C) [7].

KOH solutions with concentrations of 1, 2, and 3 mM were prepared with a final volume of 1 L each. Then, 5.0 g XG and 2.0 g KCl were added to the different KOH solutions and stirred at 25 °C for 3 h to complete dissolution. Subsequently, the pH of the solution was adjusted to 7.0. The derivatives were precipitated with anhydrous ethanol (1:4, *v*/*v*) and dried at 50 °C for 4 h. The resulting material was ground into a powder and passed through an 80-mesh sieve. Based on the KOH concentration, the obtained samples were named XG-A1 (1 mM), XG-A2 (2 mM), and XG-A3 (3 mM) [5].

After ethanol precipitation and drying, the recoveries of the enzymatically hydrolyzed, oxalic acid-treated, and dilute alkali-treated xanthan gum samples were 76.38 ± 4.12%, 78.56 ± 3.45% and 79.15 ± 3.21%, respectively.

### 3.4. Monosaccharide Composition Analysis

The monosaccharide composition of the samples was determined using an Agilent 1260 Infinity II high-performance liquid chromatography (HPLC) system (Agilent Technologies Inc., Santa Clara, CA, USA), according to a previously established method [49]. Briefly, 4 g/L XG solution was prepared using deionized water. A 0.5 mL aliquot of the sample solution was mixed with an equal volume of 2 M trifluoroacetic acid and hydrolyzed at 120 °C for 2 h. The hydrolyzed sample was dried under nitrogen stream and reconstituted with 1 mL deionized water. Then, 50 μL of the reconstituted sample was mixed with 50 μL of 0.6 M NaOH and 100 μL of 0.5 M 1-phenyl-3-methyl-5-pyrazolone methanol solution. The mixture was reacted in a 70 °C water bath for 100 min, then cooled to room temperature. Subsequently, 100 μL of 0.3 M HCl was added for neutralization, and deionized water added to a final volume of 1 mL. The mixture was extracted with 1 mL chloroform, and the organic phase discarded; this extraction process was repeated three times. The upper aqueous phase was filtered through a 0.22 μm polyethersulfone membrane to obtain the solution used for monosaccharide composition analysis. Monosaccharide standards (0.5 mg/mL) and a blank were processed using the same method described above. Monosaccharide composition was analyzed via HPLC with the following parameters: Hypersil GOLD column (250 × 4.6 mm, 8 μm; Thermo Fisher Scientific, Waltham, MA, USA), mobile phase consisting of phosphate buffer (0.1 M, pH 6.7) and acetonitrile (86:14, *v*/*v*), column temperature of 30 °C, UV detector wavelength of 245 nm, flow rate of 0.8 mL/min, and injection volume of 5 μL.

### 3.5. Molecular Weight Determination

The molecular weights of various XG derivative samples were determined via gel permeation chromatography (GPC), according to a previously established method [50]. Briefly, XG solutions with a concentration of 0.5 g/L were prepared using deionized water, centrifuged at 10,000 rpm for 10 min to remove impurities, and filtered through a 0.22 μm polyethersulfone membrane to prepare the samples. Molecular weight was measured using an Agilent 1260 Infinity II GPC/SEC system (Agilent Technologies Inc.) equipped with a PL aquagel-OH MIXED-H column (300 × 7.5 mm, 8 μm; Agilent Technologies). The mobile phase comprised 1 g/L NaNO3 solution, with a flow rate of 1 mL/min, column temperature of 30 °C, and injection volume of 100 μL.

### 3.6. ATR-FTIR and XRD Analyses

After collecting the background spectrum, a small amount of the sample was placed on a reflective crystal, and the sample spectrum then collected (NicoletiS10; Thermo Fisher Scientific). The scan was carried out at a range of 600–4000 nm, resolution of 4 cm^−1^, and 32 scans per sample [51].

The XRD patterns of various XG samples were measured at 25 °C using a Smartlab SE X-ray diffractometer (Rigaku Corporation, Tokyo, Japan). The measurement was carried out at Cu Kα radiation, 40 kV, 40 mA, scan speed of 10°/min, and scan angle (2θ) of 5–60° [52].

### 3.7. Zeta Potential

Zeta potential (ZP) values of the samples were determined using a Zetasizer Nano ZS nanosizer and ZP analyzer (Malvern Panalytical Ltd., Worcestershire, UK), according to a previously reported method [53]. Prior to the measurements, the samples were diluted with deionized water to a concentration of 0.1 mg/mL. For the ZP measurements, three replicate readings were recorded for each sample.

### 3.8. Viscosity Measurements

Viscosity of the samples was determined using a DV-2T rotary viscometer (Brookfield Engineering Laboratories, Inc., Middleboro, MA, USA). Solutions containing 1% sample and 1% KCl were prepared and continuously stirred at 25 °C and 800 rpm for 2 h. The No. 3 (0–2000 cP) and No. 4 (0–10,000 cP) rotor models were used. The sample viscosity was measured at 60 rpm for 1 min, and the values subsequently recorded.

### 3.9. Gel Preparation

Mixed gels were prepared according to a previously reported method [54], with slight modifications. Briefly, LBG and XG were accurately weighed and separately dissolved at a mass ratio of LBG to XG of 3:7, and the total polysaccharide concentration was 1.0%. This specific ratio and concentration were selected based on the references and preliminary experiments, as they exhibited the most stable synergistic gelation behavior, which is optimal for evaluating the structure–function relationships. After the mixed solution was completely dissolved, it was stirred at 80 °C and 800 rpm for 90 min and then cooled to room temperature to obtain the XG–LBG composite gel. The gel samples were stored at 4 °C for 12 h before testing.

### 3.10. Thermal Stability Analysis

To eliminate the interference of water evaporation and focus on the thermal decomposition behavior of the composite gel network, thermogravimetric analysis was performed on lyophilized gels with slight modifications according to a previously established method [46,55], 3 mg of the freeze-dried gel sample was placed in an aluminum crucible and heated from 25 °C to 600 °C at a rate of 10 °C/min under a nitrogen flow rate of 20 mL/min (SDT Q600; TA Instruments, New Castle, DE, USA).

### 3.11. Rheological Property Measurements

Static and dynamic rheological properties of the XG–LBG gels were measured using an MCR 302 rheometer (Anton Paar GmbH, Graz, Austria), according to a previously reported method [56]. The rheometer was equipped with a 25 mm parallel plate geometry with a fixed gap of 1 mm. Gel samples (diameter: 25 mm, thickness: 1 mm) were used. The strain was adjusted from 0.1% to 1000% at a frequency of 1 Hz to determine the linear viscoelastic region (LVR); a strain of 1% was used in subsequent experiments. Frequency sweep experiments were conducted in the range of 0.1–10 Hz, recording the storage modulus (*G′*), loss modulus (*G″*), and loss tangent (*tanδ*). The gelation and melting temperatures of XG–LBG solutions were investigated using temperature sweep experiments. The sample temperature was increased from 5 °C to 80 °C, held at 80 °C for 10 min, and then cooled to 5 °C. Both the heating and cooling rates were 5 °C/min, and the frequency was 1 Hz. To prevent solvent evaporation, the periphery of the sample was covered with silicone oil.

### 3.12. Texture Analysis

Textural properties of the mixed gels were measured according to a previously reported method [57], with slight modifications. The textures of XG–LBG composite gels were analyzed at room temperature using a CT3 1500 texture analyzer (Brookfield Engineering Laboratories, Inc.) equipped with a TA 10 probe in compression test mode. The gel samples were prepared as cylinders with a diameter of 5.0 cm and height of 4.0 cm. The test speed was 1.5 mm/s. The compression ratio, hold time, and trigger force were set to 50%, 3 s, and 5 g, respectively.

### 3.13. LF-NMR Analysis

The transverse relaxation time (T_2_) of the composite gel samples was determined using an LF-NMR analyzer (MesoMR23-040V; Niumag Corporation, Shanghai, China), according to a previously reported method [58]. XG–LBG composite gels were prepared according to the method described in Section 2.3. The mixed solution (35 mL) was transferred into a cylindrical NMR glass tube (diameter: 40 mm). After gel formation, the glass tube was inserted into the NMR analyzer, and the T_2_ analyzed using the Carr–Purcell–Meiboom–Gill sequence. The parameter settings were as follows: sampling bandwidth = 200 kHz, regulate analog gain = 20 dB, number of echoes = 18,000, echo time = 0.15 ms, number of scans = 8, and time wait = 8000 ms.

### 3.14. Scanning Electron Microscope

The morphology of XG–LBG gels was investigated using a SUPRA 55 thermal field emission scanning electron microscope (Carl Zeiss AG, Oberkochen, Germany), according to a previously reported method [59]. The samples were frozen in liquid nitrogen and subsequently freeze-dried. The freeze-dried samples were fixed onto the sample stage with conductive adhesive, followed by sputter-coating with gold before observation. Images were recorded at magnifications of 100× and 200× under an accelerating voltage of 5 kV. Porosity and pore size distribution were quantified from the SEM images using ImageJ software (v.1.8.0, National Institutes of Health, Bethesda, MD, USA). For each sample, three representative micrographs were converted to 8-bit grayscale, automatically segmented into pore and solid regions using the Otsu thresholding method and the area fraction of pores (porosity, %) mean pore size (μm) and the standard deviation (SD)were calculated using the Analyze Particles function.

### 3.15. Statistical Analysis

All experiments were performed in triplicate, and the data reported as mean ± standard deviation (SD). Statistical analyses were performed using SPSS v.26.0 (IBM, Armonk, NY, USA) and Origin 2021 (Originlab Corporation, Northampton, MA, USA). Heatmap analysis was conducted using Origin 2021 based on the Pearson correlation coefficient. A *p*-value < 0.05 was considered to indicate statistically significant differences.

## 4. Conclusions

In this study, three targeted modification strategies (enzymatic hydrolysis, oxalic acid treatment, and dilute alkali treatment) were applied to precisely regulate the side chain groups of XG, and the regulatory mechanism of XG fine structure on XG/LBG composite gel properties was systematically elucidated via multi-scale characterization. Results showed that pyruvate and glucuronic acid contents exhibited an extremely significant positive correlation with gel strength and *G′*, enhancing network stability through electrostatic interactions and hydrogen bonding. Acetyl groups inhibited interchain hydrogen crosslinking via steric hindrance, while moderate deacetylation significantly increased gel hardness and *G′* by exposing side chain binding sites. Pyruvate removal endowed gels with optimal thermal stability, whereas excessive enzymatic hydrolysis markedly weakened gel network strength. Multi-scale characterizations further corroborated the correlation between XG side chain structure, gel microstructure and macroscopic performance. This study clarified the distinct functional roles of key XG side chain groups in the synergistic gel system, deepened the understanding of XG-LBG synergistic gelation, and provided critical theoretical support for the precise design of XG-based functional composite gels.

## Figures and Tables

**Table 1:** Determination of pyruvate content, acetyl content, molecular weight, viscosity and monosaccharide composition of different modified XG.

Sample	Pyruvate Content (%)	Acetyl Content (%)	Mw × 10^7^ (Da)	Viscosity (cP)	Molar Ratios (mol %)		
					Glucose	Mannose	Glucuronic Acid
XG	5.80 ± 0.42 ^a^	3.97 ± 0.38 ^a^	2.20 ± 0.29 ^a^	1654 ± 21 ^d^	52.46 ± 0.91 ^d^	38.74 ± 2.11 ^ab^	8.80 ± 1.35 ^a^
XG−HPM	4.81 ± 0.13 ^b^	4.03 ± 0.11 ^a^	2.07 ± 0.08 ^a^	1713 ± 17 ^c^	56.76 ± 1.40 ^c^	40.42 ± 2.02 ^a^	2.82 ± 0.69 ^b^
XG−MPM	3.15 ± 0.11 ^d^	4.03 ± 0.14 ^a^	1.72 ± 0.02 ^b^	1908 ± 13 ^b^	60.05 ± 1.83 ^b^	38.54 ± 2.24 ^ab^	1.41 ± 0.42 ^bc^
XG−LPM	1.05 ± 0.11 ^e^	4.13 ± 0.27 ^a^	1.59 ± 0.05 ^b^	2390 ± 26 ^a^	64.10 ± 1.87 ^a^	34.90 ± 2.10 ^c^	1.00 ± 0.29 ^c^
XG−HP	4.92 ± 0.17 ^b^	3.17 ± 0.14 ^b^	1.17 ± 0.05 ^cd^	1381 ± 11 ^f^	53.00 ± 0.94 ^d^	37.64 ± 1.62 ^abc^	9.36 ± 0.68 ^a^
XG−MP	3.85 ± 0.02 ^c^	2.98 ± 0.08 ^bc^	0.92 ± 0.01 ^e^	1430 ± 26 ^f^	52.86 ± 0.37 ^d^	37.71 ± 0.91 ^abc^	9.43 ± 0.55 ^a^
XG−LP	1.42 ± 0.01 ^e^	2.51 ± 0.12 ^d^	0.84 ± 0.07 ^e^	1418 ± 7 ^f^	53.13 ± 0.28 ^d^	37.39 ± 0.92 ^abc^	9.48 ± 0.64 ^a^
XG−A1	5.77 ± 0.06 ^a^	3.38 ± 0.08 ^b^	1.27 ± 0.11 ^c^	1499 ± 24 ^e^	52.62 ± 0.27 ^d^	38.12 ± 1.12 ^abc^	9.26 ± 0.85 ^a^
XG−A2	6.12 ± 0.07 ^a^	3.08 ± 0.13 ^b^	1.22 ± 0.05 ^c^	1480 ± 3 ^e^	52.85 ± 0.56 ^d^	36.96 ± 1.19 ^bc^	10.18 ± 0.63 ^a^
XG−A3	6.06 ± 0.16 ^a^	2.58 ± 0.12 ^cd^	0.96 ± 0.08 ^de^	1479 ± 10 ^e^	53.39 ± 0.73 ^d^	37.04 ± 0.57 ^bc^	9.57 ± 0.17 ^a^

Note: Different lowercase letters in the same column indicate significant difference (*p* < 0.05). In the sample codes, “XG” stands for commercial xanthan gum as the control group. “HPM”, “MPM”, and “LPM” respectively represent high, medium, and low levels of pyruvated mannan content in the side chains of xanthan gum, while “HP”, “MP”, and “LP” respectively represent high, medium, and low levels of pyruvic acid content. “A1”, “A2”, and “A3” respectively represent low, moderately low, and moderate degrees of acetyl group removal.

**Table 2:** Determination of texture properties of composite gels.

Sample	Hardness (g)	Cohesiveness	Elasticity (mm)	Adhesiveness (g)	Chewiness (mJ)
XG/LBG	280.47 ± 9.30 ^bc^	0.29 ± 0.04 ^cd^	22.41 ± 0.30 ^ab^	82.10 ± 11.61 ^ab^	18.02 ± 2.34 ^a^
HPM/LBG	183.07 ± 15.01 ^d^	0.29 ± 0.06 ^cd^	22.80 ± 0.82 ^ab^	53.27 ± 12.27 ^cd^	12.01 ± 2.77 ^cd^
MPM/LBG	122.20 ± 13.23 ^e^	0.33 ± 0.04 ^c^	21.56 ± 0.65 ^ab^	39.87 ± 6.45 ^d^	8.41 ± 1.12 ^d^
LPM/LBG	32.20 ± 2.25 ^f^	0.60 ± 0.05 ^a^	17.92 ± 1.03 ^c^	19.30 ± 2.52 ^e^	3.37 ± 0.24 ^e^
HP/LBG	246.77 ± 27.09 ^c^	0.25 ± 0.01 ^d^	21.49 ± 0.48 ^b^	66.90 ± 4.23 ^bc^	13.80 ± 0.56 ^bc^
MP/LBG	166.73 ± 4.15 ^d^	0.33 ± 0.01 ^c^	21.98 ± 0.76 ^ab^	50.47 ± 8.55 ^d^	16.72 ± 3.06 ^ab^
LP/LBG	153.60 ± 6.69 ^e^	0.40 ± 0.03 ^b^	21.93 ± 1.09 ^ab^	55.37 ± 0.21 ^cd^	11.94 ± 0.40 ^cd^
A1/LBG	262.73 ± 31.29 ^c^	0.34 ± 0.01 ^c^	22.91 ± 0.59 ^a^	88.67 ± 7.95 ^a^	19.87 ± 2.17 ^a^
A2/LBG	309.13 ± 22.96 ^ab^	0.28 ± 0.01 ^cd^	22.40 ± 0.41 ^ab^	86.77 ± 8.47 ^a^	19.03 ± 1.50 ^a^
A3/LBG	325.87 ± 14.29 ^a^	0.27 ± 0.01 ^cd^	22.59 ± 0.13 ^ab^	88.97 ± 6.36 ^a^	19.73 ± 1.33 ^a^

Note: Different lowercase letters in the same column indicate significant difference (*p* < 0.05). In the sample codes, “XG” stands for commercial xanthan gum as the control group. “HPM”, “MPM”, and “LPM” respectively represent high, medium, and low levels of pyruvated mannan content in the side chains of xanthan gum, while “HP”, “MP”, and “LP” respectively represent high, medium, and low levels of pyruvic acid content. “A1”, “A2”, and “A3” respectively represent low, moderately low, and moderate degrees of acetyl group removal. “LBG” indicates that the XG sample is mixed with LBG.

**Table 3:** Pore indices of different gel samples.

Sample	Porosity (%)	Pore Size (μm)	Standard Deviation (μm)
XG/LBG	67.11 ± 3.80	221.24	32.76
HPM/LBG	74.49 ± 4.17	240.75	34.12
MPM/LBG	78.89 ± 4.73	268.31	38.15
LPM/LBG	88.17 ± 5.29	308.16	36.95
HP/LBG	66.20 ± 4.71	189.02	42.34
MP/LBG	67.07 ± 3.82	230.55	41.95
LP/LBG	77.99 ± 4.61	241.74	42.21
A1/LBG	64.34 ± 3.21	196.23	31.97
A2/LBG	59.44 ± 3.47	120.45	24.83
A3/LBG	50.75 ± 2.79	101.48	30.91

## Data Availability

The raw data supporting the conclusions of this article will be made available by the authors on request.
